# Grouping and Read-Across Approaches for Risk Assessment of Nanomaterials

**DOI:** 10.3390/ijerph121013415

**Published:** 2015-10-26

**Authors:** Agnes G. Oomen, Eric A. J. Bleeker, Peter M. J. Bos, Fleur van Broekhuizen, Stefania Gottardo, Monique Groenewold, Danail Hristozov, Kerstin Hund-Rinke, Muhammad-Adeel Irfan, Antonio Marcomini, Willie J. G. M. Peijnenburg, Kirsten Rasmussen, Araceli Sánchez Jiménez, Janeck J. Scott-Fordsmand, Martie van Tongeren, Karin Wiench, Wendel Wohlleben, Robert Landsiedel

**Affiliations:** 1 National Institute for Public Health and the Environment (RIVM), PO Box 1, Bilthoven 3720, The Netherlands; E-Mails: Eric.Bleeker@rivm.nl (E.A.J.B.); Peter.Bos@rivm.nl (P.M.J.B.); Fleur.van.Broekhuizen@rivm.nl (F.B.); Monique.Groenewold@rivm.nl (M.G.); Willie.Peijnenburg@rivm.nl (W.J.G.M.P.); 2 Joint Research Centre, European Commission, Via E. Fermi 2749, Ispra 21027, Italy; E-Mails: Stefania.GOTTARDO@ec.europa.eu (S.G.); Kirsten.RASMUSSEN@ec.europa.eu (K.R.); 3 Department of Environmental Sciences, Informatics and Statistics, University Ca’ Foscari of Venice, Vegapark, Via delle Industrie 21/8, Marghera 30175, Venice, Italy; E-Mails: Danail.Hristozov@unive.it (D.H.); marcom@unive.it (A.M.); 4 Fraunhofer Institute for Molecular Biology and Applied Ecology, Auf dem Aberg 1, Schmallenberg 57392, Germany; E-Mail: Kerstin.Hund-Rinke@ime.fraunhofer.de; 5 BASF SE, GB/TB-Z470, Ludwigshafen 67056, Germany; E-Mails: muhammad-adeel.irfan@basf.com (M.-A.I.); karin.wiench@basf.com (K.W.); wendel.wohlleben@basf.com (W.W.); robert.landsiedel@basf.com (R.L.); 6 Centre for Environmental Sciences, University Leiden, PO Box 9518, Leiden 2300, The Netherlands; E-Mail: peijnenburg@cml.leidenuniv.nl; 7 Centre for Human Exposure Science (CHES), Institute of Occupational Medicine (IOM), Edinburgh EH14 4AP, UK; E-Mails: Araceli.Sanchez@iom-world.org (A.S.J.); Martie.VanTongeren@iom-world.org (M.T.); 8 Dept Bioscience, Aarhus University, Vejlsøvej 25, PO Box 314, Silkeborg 8600, Denmark; E-Mail: jsf@dmu.dk

**Keywords:** nanomaterials, grouping, read-across, MARINA risk assessment strategy

## Abstract

Physicochemical properties of chemicals affect their exposure, toxicokinetics/fate and hazard, and for nanomaterials, the variation of these properties results in a wide variety of materials with potentially different risks. To limit the amount of testing for risk assessment, the information gathering process for nanomaterials needs to be efficient. At the same time, sufficient information to assess the safety of human health and the environment should be available for each nanomaterial. Grouping and read-across approaches can be utilised to meet these goals. This article presents different possible applications of grouping and read-across for nanomaterials within the broader perspective of the MARINA Risk Assessment Strategy (RAS), as developed in the EU FP7 project MARINA. Firstly, nanomaterials can be grouped based on limited variation in physicochemical properties to subsequently design an efficient testing strategy that covers the entire group. Secondly, knowledge about exposure, toxicokinetics/fate or hazard, for example via properties such as dissolution rate, aspect ratio, chemical (non-)activity, can be used to organise similar materials in generic groups to frame issues that need further attention, or potentially to read-across. Thirdly, when data related to specific endpoints is required, read-across can be considered, using data from a source material for the target nanomaterial. Read-across could be based on a scientifically sound justification that exposure, distribution to the target (fate/toxicokinetics) and hazard of the target material are similar to, or less than, the source material. These grouping and read-across approaches pave the way for better use of available information on nanomaterials and are flexible enough to allow future adaptations related to scientific developments.

## 1. Introduction

Nanomaterials (NMs) have many physicochemical properties that can affect their exposure, toxicokinetics/fate and hazard [1,2,3,4,5,6]. NMs that have slightly different physicochemical properties, for example a small variation in the surface characteristics or in the size distribution, may potentially have significantly different risk profiles. At present, the knowledge on relationships between such variation in physicochemical properties and variation in effects of NMs is limited [7], and systematic gathering of information across NMs is therefore encouraged. At the same time, there is a high, and challenging, need for efficient approaches aimed to information gathering for risk assessment, which should ensure that sufficient information is available to assess the safety of NMs while minimising the need for animal testing. This urgency is felt by industry and regulatory authorities alike, as the costs to warrant safety by extensive testing of each slightly different NM, and to assess each material, could be exorbitant and could hamper innovation in the field. Furthermore, extensive *in vivo* testing would challenge the international ethically based intention to reduce animal testing.

Approaches supporting the risk assessment of NMs should be applicable at present, as well as sufficiently flexible to allow incorporation of future scientific developments relating to knowledge on NM behaviour and material innovation. Such approaches include risk assessment strategies, identification of generic groups to guide efficient testing of NMs, and read-across between nano and non-nanomaterials.

A Risk Assessment Strategy (RAS) that addresses specific challenges for NMs, including how to perform risk assessment for the significant number of NMs currently in development or already marketed, as well as dealing with the changes in physicochemical properties of NMs during their life cycle, is developed in the context of the EU-funded FP7 project MARINA (http://www.marina-fp7.eu/). The MARINA RAS is designed to use the scientific insights on NMs and provide a generic framework, allowing it to be considered and embraced by other projects and organisations. The outline of the MARINA RAS is described by Bos *et al*. [8]. One of the challenges in developing efficient risk assessment approaches for NMs is to make best use of the-still scarce-available information and data on physicochemical properties, exposure, toxicokinetics, fate, and hazard. Grouping and read-across approaches for NMs can help to streamline this best use of information.

## 2. Aim

The aim of the present manuscript is to describe the integration of grouping and read-across approaches in the NM-tailored MARINA RAS. This should serve the overall purpose of efficient information gathering for risk assessment of NMs in a way that ensures that safety can be assessed.

## 3. Terminology

For clarity, the terms *group* and *read-across* are defined for the purpose of this paper.

A *group* represents a number of NMs which share a commonality relevant for risk, which can be one or more common property(ies) in a physical, chemical, exposure, (eco)toxicological, toxicokinetics or fate sense. A NM can belong to more than one group.

*Read-across* refers to predicting hazard information on one or more endpoints relevant for risk assessment of one NM or more NMs (target material(s)) by using data from another NM or more NMs (source material(s)). The source materials can also be non-nano.

In a regulatory context, the OECD (Organisation for Economic Co-operation and Development) [9] has issued general guidance on grouping and read-across via the document “Guidance on Grouping of Chemicals, Second Edition”, available at http://www.oecd.org/chemicalsafety/risk-assessment/groupingofchemicalschemicalcategoriesandread-across.htm. Furthermore, in the EU, the European Chemicals Agency (ECHA) has defined read-across for chemicals in general [10] as a technique for predicting endpoint information for one substance (target substance) by using data for the same endpoint from another substance or other substances (source substances). As this definition was developed before NMs were considered in regulations, read-across refers to predicting information from one substance to another. In the present paper, the term read-across also refers to data sharing between NMs and/or non-NMs of the same substance, for example from one specific (nano) form of titanium dioxide to another specific (nano) form of titanium dioxide.

## 4. Key Elements

In the present paper, the application of approaches for grouping and read-across are explored for NMs. To that end, five key elements that need to be taken into account are discussed.

A first key element is that grouping and read-across for NMs should be useful approaches in a regulatory framework or strategy for risk assessment. The present concepts for grouping and read-across for NMs are put into the broader scope of the MARINA RAS [8]. Furthermore, the RAS should allow application of grouping and read-across for NMs now and be sufficiently flexible to accommodate future increases in knowledge and material development.

A second key element is that proposals for grouping and read-across of NMs should be based on a solid scientific justification. This is required for chemicals in general under the EU REACH legislation (Regulation (EC) No 1907/2006), recommended in the OECD guidance on general principles for grouping [9], and also indicated by the ECHA Group Assessing Already Registered Nanomaterials (GAARN) [11].

**Figure 1:**
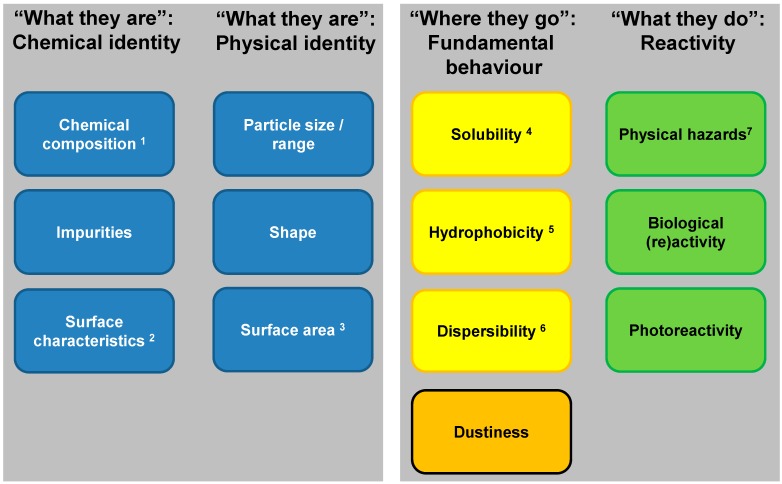
Key properties that characterize a NM (adapted from Seller *et al.* [1]), arranged under headings from ITS-NANO [5], and coloured in accordance with Figure 2. These properties can affect exposure, toxicokinetics, fate and/or (eco) toxicological behaviour and thus the risk posed by NMs, and constitute the basic information needed (based on current knowledge) to implement the assessment in Figure 3, Figure 4 and Figure 5. The information on chemical and physical identity (“What they are”) can be used for a first comparison of a certain NM to other NMs. Note that (i) some properties may not be relevant for all NMs, for example dustiness only applies to powders; (ii) some properties (for example dispersibility, dustiness) are system-dependent properties, and (iii) the key physicochemical properties are based on the present knowledge derived mostly from studying NMs that are metals, metal oxides or carbon based. Legend for the superscript numbers providing additional information on the properties: 1. Chemical composition comprises crystal structure; 2. Surface characteristics, which includes coating chemistry, functionalization (e.g., capping agents), surface charge (e.g., zeta potential); 3. Surface area, which includes porosity; 4. Solubility includes water equilibrium solubility and rate of dissolution in relevant media; 5. Hydrophobicity for NMs is dependent on e.g., van der Waals energy, Hamaker constant, zeta potential. Analytical determination of the hydrophobicity of NMs is still under development, e.g., sessile drop contact angle, dye adsorption; 6. Dispersibility refers to the relative number or mass of particles in a suspending medium, and relates to stability [1], aggregation and agglomeration in relevant media, and is dependent on e.g., van der Waals energy, Hamaker constant, zeta potential; 7. Physical hazards comprise explosiveness, flammability, and autoflammability.

A third element is that the NMs considered should be unequivocally identified and characterized. This information is used (1) to assess what information on the specific NM is already available, and (2) for risk assessment, including grouping and read-across options. Without this information to characterize the NMs, these processes cannot be initiated. Several initiatives propose similar sets of physicochemical properties to characterize NMs [1,11,12,13,14]. Figure 1 presents a set of physicochemical properties that can affect exposure, toxicokinetics, fate and/or (eco)toxicological behaviour, and is based on Sellers *et al.* [1]. As is the case for substances in general, impurities in NMs may also contribute to or determine the toxicity and should therefore be identified. The properties are sub-divided into four classes that were proposed by Stone *et al.* [5]: “What they are”: Chemical identity, “What they are”: Physical identity, “Where they go”: Fundamental behaviour, and “What they do”: Reactivity. Hence, as each specific NM can have a different risk profile, the current knowledge indicates that information on these key properties needs to be considered. The properties related to chemical and physical identity can be used to support a first comparison of the specific NM to other NMs.

**Figure 2:**
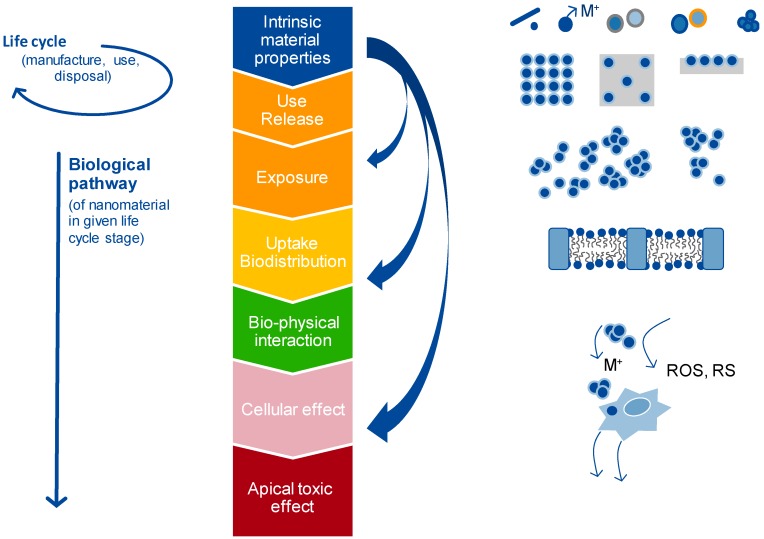
A sequence of events in the life cycle and the biological pathways that can influence the apical effect (adapted from Arts *et al.* [2], Arts *et al.* [3], Oomen *et al.* [4]). Material physicochemical properties (blue) affect exposure (orange), toxicokinetics (yellow, green) and hazards (pink, red) in potentially different ways, and thereby influence the apical effect. This should be taken into consideration in grouping and read-across approaches

A fourth element is that, as described by Oomen *et al.* [4] and Arts *et al.* [2,3], a sequence of events takes place between NM production and the potential occurrence of a toxic effect, as illustrated in Figure 2. Physicochemical properties affect the whole life cycle of the material including the biological pathway in potentially different ways. It is thus key that this is taken into account for grouping and read-across among NMs. Here, this is addressed by the option to consider the influence of changes in physicochemical properties on exposure, toxicokinetics and hazard, separately, followed by integration for assessment purposes.

A final element relates to the mechanistic toxicological pathways. It has been discussed by several authors (e.g., Nel *et al.* [15], Donaldson and Poland [16] and Gebel *et al.* [17]) that there seem to be no new modes of action for NMs compared to the modes of action already known for non-nano materials. Moreover, in contrast to non-nano materials, the number of toxicological mechanistic pathways for NMs seems to be limited to a handful [2,15,17], and may be related to the limited potential of the (predominantly investigated metal, metal oxide and carbon based) NMs for chemically-based receptor-binding. This element may streamline and simplify grouping and read-across for a target NM, and application of these nano-specific insights in the MARINA RAS should be possible.

## 5. Existing Concepts for Grouping and Read-Across of NMs

The European Centre for Ecotoxicology and Toxicology of Chemicals (ECETOC) Task Force on the Grouping of Nanomaterials recently reviewed the available concepts for the grouping of NMs for human health risk assessment [3], which is referred to for a more detailed overview of existing concepts for grouping. Briefly, grouping concepts for nanomaterials have also been developed by the British Standards Institute (https://nanohub.org/groups/gng/guidelines), the German Federal Institute for Occupational Safety and Health [18], Kuempel *et al.* from US NIOSH [19], and Gebel *et al.* [17]. As discussed by Gebel *et al.* [17] and Arts *et al.* [3] these concepts are generally consistent with each other. These groups are referred to by Gebel *et al.* [17] as a group with “chemically mediated toxicity”, “toxicity caused by certain fibrous NMs”, and “toxicity through granular biodurable nanoparticles”. In later publications, these groups were referred to as “active NMs”, “biopersistent high aspect ratio (HAR) NMs” and “passive NMs” respectively, and supplemented by a group with “soluble NMs” [2]. Godwin *et al.* [20] also summarized approaches to grouping and ranking NMs, differentiating by categorization according to “physicochemical properties”, “exposure and use scenarios” and “linking selected physicochemical properties to specific biological outcomes”.

It should be noted that these concepts generally describe broad groups of NMs and focus mainly on human health hazards via the inhalation exposure route. For a considerable number of NMs, grouping according to these proposals will not enable risk assessment-based decisions, as information may be too limited to draw clear conclusions and because these groups may still be too heterogeneous with regard to the various physicochemical properties. Information from similar materials as in these grouping proposals can aid in highlighting those issues that need specific attention in risk assessment. In some cases, it may be possible to identify a group of NMs for which read-across early in the risk assessment process is feasible. For example, read-across to the solute of quickly dissolving NMs may be possible. In other cases, there is a need to identify under which circumstances data from certain materials in the group can be used to fill in data gaps of a new material endpoint-by-endpoint.

## 6. Grouping and Read-Across among NMs within the MARINA Risk Assessment Strategy

**Figure 3:**
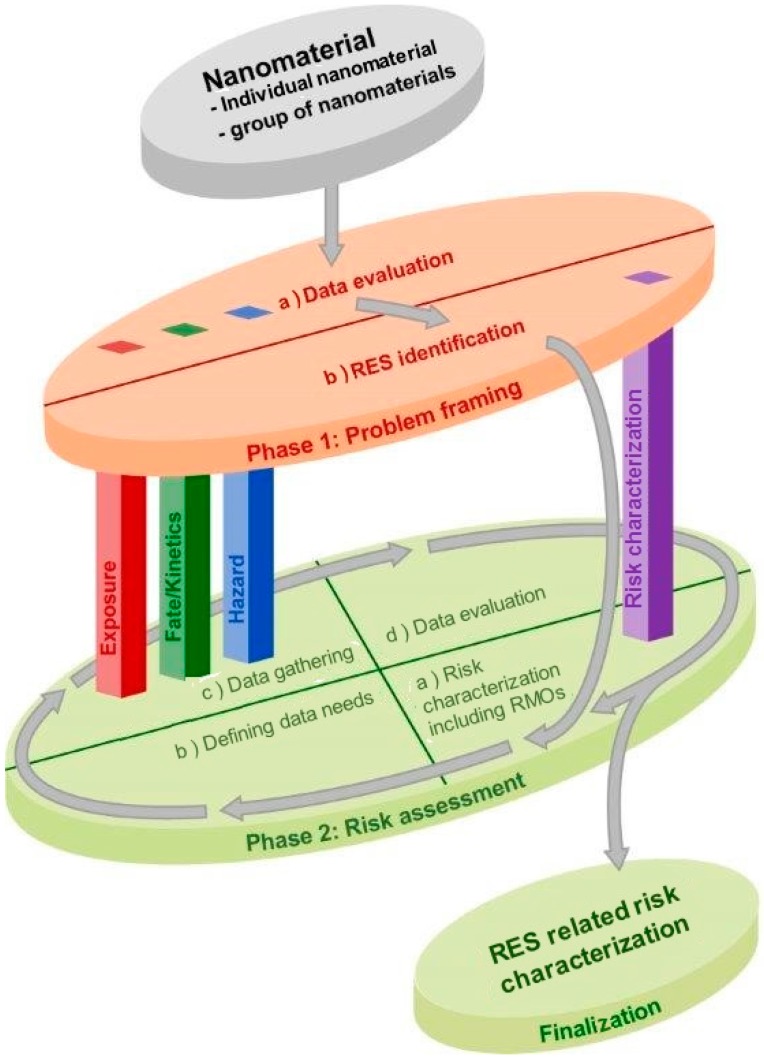
Schematic overview of the MARINA Risk Assessment Strategy, consisting of an overarching Phase 1: Problem Framing (orange), and the iterative Phase 2: Risk Assessment (green) with a cyclic evaluation process. The information gathering is organised in three pillars (Exposure, Fate/Toxicokinetics and Hazard). The integration of the gathered information to derive a conclusion on risk is represented by a separate pillar *i.e.*, Risk characterization. Each pillar comprises tools both for Phases 1 and 2 (from Bos *et al.* [8]). RES: Relevant Exposure Scenario. RMO: Risk Management Options.

**Figure 4:**
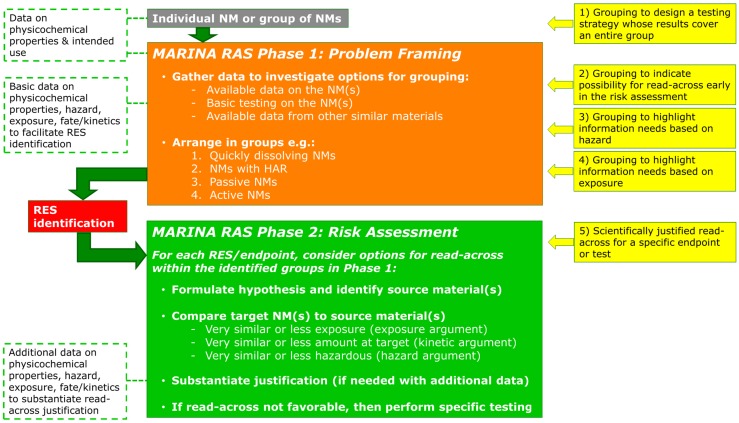
Possible applications of grouping and read-across of NMs (yellow boxes) in Phase 1 (orange box) and 2 (green box) of the MARINA Risk Assessment Strategy (RAS). These boxes are explained in more detail in the text in order of their numbering. The white boxes with green dotted outline represent input of data. HAR: High Aspect Ratio; RES: Relevant Exposure Scenario.

In the present paper, the integration of approaches for grouping and read-across among NMs into the MARINA RAS is discussed. This strategy is composed of two phases: “Phase 1: Problem Framing” and “Phase 2: Risk Assessment” (see Figure 3 and Figure 4). The aim of Phase 1 is to obtain a basic view of the potential risk of the NM by identifying Relevant Exposure Scenarios (RESs) throughout an NM’s life cycle and verifying whether exposure in these scenarios may potentially lead to adverse health and/or environmental effects. “Relevant” in this context means important from the viewpoint of exposure, fate, toxicokinetics and/or hazard, and thus demanding further evaluation in Phase 2. The Problem Framing Phase should (i) define the scope of Phase 2 by evaluating all available information, including a set of basic requirements, and defining information and/or testing requirements to be addressed in Phase 2, and (ii) provide guidance for the strategy to collect this information. More details on the MARINA RAS can be found in Bos *et al.* [8].

According to the MARINA RAS, information on key physicochemical properties of a NM should be available to support Phase 1. Furthermore, in Phase 1 basic exposure information, at least based on the intended use (which includes physical form and anticipated exposure route(s)), and basic toxicokinetic and hazard information need to be available. Information from similar materials and applications may be used. This includes exposure information related to the physical form (e.g., powder, suspension) in combination with similar applications and/or processes. This kind of information is for example gathered in the MARINA exposure scenario library for NMs [21], or related to qualifiers on use, release and route of exposure as indicated in Arts *et al.* [2]. By combining physicochemical, exposure, kinetic and hazard information, RESs are identified for further consideration in Phase 2 of the strategy. In some cases, these elements of basic information may indicate already in Phase 1 that risks (for some uses) are expected to be high, which should be considered in further development and/or application of the NM under consideration. By identifying a likely risk, a number of measures can be considered, including avoiding specific exposure routes via modification (or withdrawal) of the material/product, risk management measures such as application of safer designs or personal protective equipment. The time and costs needed to obtain all information required for risk assessment should be evaluated taking into account also potential benefits and profits and the likelihood of regulatory acceptance after further testing. For example, the information, of physicochemical nature, that a material is an HAR material, can be an alert for a possible specific human health hazard (asbestos-like effects), and the potential application of this material should be carefully considered based on this information.

Several different possibilities for application of grouping and read-across in the two Phases of the MARINA RAS are illustrated in Figure 4; the numbers of the following sections correspond to the numbers in Figure 4.

### 6.1. Ad One: Grouping to Design A Testing Strategy Whose Results Cover An Entire Group of NMs

This application of grouping is a pragmatic approach where it is not an individual NM that “enters” the MARINA RAS, but a group of NMs. In this case, data for risk assessment purposes can be gathered in an efficient way by testing a few selected NMs in the group and interpolate test results to cover the entire group. To that end, a group of NMs needs to be identified before testing is performed, *i.e.*, prior to or at the start of Phase 1 in the MARINA RAS. A proposal for grouping is mainly based on limited variation in physicochemical properties of NMs and their intended use (this input information is indicated in upper white box with green dotted outline in Figure 4). The physicochemical properties that should be available from the beginning for each NM are listed in the chemical and physical identity columns in Figure 1, and are used to determine if and what information on the investigated NMs is already available, and to compare the NMs to conclude whether they can form a group for risk assessment purposes. In addition, intended use(s) should be considered in the group proposal as this is associated with the physical form, relevant exposure routes and exposure potential. Selected NMs of the proposed group can subsequently be tested according to the MARINA RAS so that the outcome of the testing would cover the entire group by interpolation on an endpoint basis (*i.e.*, in Phase 2 of the MARINA RAS). To that end, at least the NMs that define the borders of variation in physicochemical property/ies are investigated, as well as some NMs in between to support a certain trend between variable properties and an effect. Depending on the number of variable physicochemical properties within the group, a multi-dimensional space of several physicochemical properties will have to be described. The applicability of the test results from selected NMs to the entire group may be further substantiated by argumentation on the likelihood of trends between the variable physicochemical properties, toxicokinetics and hazard relevant for a certain endpoint based on general trends described in available scientific knowledge. The argumentation can also be strengthened with additional data, *i.e.*, physicochemical, *in vitro*, or *in vivo* data. For example, *in vitro* hazard data on a larger number of NMs from the group that confirm the relationship between the variable physicochemical property and an effect can be generated and used to substantiate the applicability of *in vivo* data on a few NMs for the entire group. In this way information for the non-tested NMs in the group can be obtained by interpolation of the test results for the NMs that define the borders of the variation in physicochemical properties and subsequent analysis of the results. Interpolation is proposed here rather than extrapolation due to the current limited understanding in behaviour and effects of NMs related to NM variation in physicochemical properties.

Finding groups of NMs for which data can be generated to cover the entire group may be challenging. In some cases, the production process may provide a starting point for formation of such a group. If the same production process is used with slightly different conditions (for example temperature and/or pressure), the size or size distribution may be different depending on these conditions, but other properties such as chemical composition, particle shape, surface properties, etcetera can be the same. These NMs can be grouped to subsequently design a testing strategy for one or more endpoints, for example according to the MARINA RAS, and whose results can cover the entire group.

Furthermore, properties that vary within the group may, to some extent, correlate. For example, size and specific surface area correlate to a certain extent, though the lowest average primary particle size does not necessarily correspond to the highest specific surface area (as for the latter the entire size distribution, shape and porosity is considered). Such correlations between physicochemical properties within a group can decrease the number of NMs that define the borders of the variation in physicochemical properties and thus reduce the number of tests that are required to cover the entire group. At present, groups of NMs with variation in properties such as chemical composition, type of surface coating and functionalization, general shape (sphere, tube, sheet, *etc*.) are generally not suitable for the type of approach described above, as the NMs cannot be arranged on a continuous scale so that the NMs that define the borders of variation in physicochemical properties cannot be identified. For variations in size, thickness or % of a specific coating surface charge et cetera the above approach is considered feasible.

After information gathering and testing in Phases 1 and 2 of the MARINA RAS, it should be considered if the proposed grouping is indeed applicable and justifiable. If the test results of the NMs, which are on the borders of variation in physicochemical properties, are very different, subgrouping may be an option. The end of Phase 1 is a first assessment opportunity on whether the proposed grouping is expected to be applicable, justifiable and useful for the intended application, and if further information gathering in Phase 2 is worthwhile.

### 6.2. Ads Two, Three and Four: Grouping That Steers The Risk Assessment Process

The groupings to indicate possibilities for read-across early in the risk assessment (ad 2), to highlight information needs based on hazard (ad 3), and to highlight information needs based on exposure (ad 4) can all be used to steer the risk assessment process based on existing knowledge on similar materials. The groups according to Arts *et al.* [2], which are similar to several other proposed groupings for NMs, can be used for steering the information gathering process in relation to certain endpoints/issues that need special attention based on experience with similar materials, e.g., experience with other quickly dissolving NMs, other NMs with HAR, other passive NMs or other active NMs. The group “soluble NMs” that is defined by Arts *et al.* [2] by a water solubility above 100 mg/l, solubility in biological media or a pulmonary half-life of less than 40 days, is adapted in this paper to a group of “quickly dissolving NMs”. For NMs in this group, it is possible to argue early in the process for read-across to data on the solute or bulk form, if this information is available. In the present publication, no definition of “quickly” will be proposed, though, clearly, the term needs further specification. For environment, “quickly” relates to the time frame for dissolution of the NM in environmentally relevant media for fate and ecotoxicity. For human health, “quickly” relates to dissolution in physiologically relevant media in relation to physiologically relevant time frames. More specifically, for the oral exposure route, the conditions relevant for transit through and uptake by the gastrointestinal tract should be considered. This concept is applied in an EFSA guidance that allows for reduced information requirements when an NM completely dissolves or degrades under conditions and in time frames relevant for the human gastrointestinal tract [22]. For inhalation exposure, the relevant time frame is determined by the time needed for dissolution in lung lining fluid combined with the time frames for removing particulate matter (*i.e.*, the time required by the mucociliary escalator to remove particulate matter from the respiratory tract, and cellular uptake of NMs from the lung lining fluid). For dermal exposure, the conditions on the skin, residence time on the skin, and translocation through the skin needs to be considered. Issues, which need to be considered for NMs that quickly dissolve, are local and systemic acute effects related to e.g., release of ions, as also indicated by Kuempel *et al.* [19].

The group of “soluble NMs” is presently adapted to “quickly dissolving NMs”, as differentiation should be made between complete dissolution before translocation across the portal of entry (*i.e.*, lung epithelium, gastrointestinal lining, skin), and partial dissolution or degradation into smaller particles. In the latter case, information of the solute cannot be automatically used, as uptake of particles can result in different distribution to organs and cells thus in different toxicity than for the solute [23]. It should be noted that information on the solubility and dissolution rate, especially in physiologically and environmentally relevant media, also provide insight into the likely biopersistence, potential for accumulation and long-term effects of a NM [24].

The identity of the solute is not always straightforward, as an NM may dissolve into different solutes. Metals, metal oxides and salts will usually dissolve into ions. The solute of other materials such as carbon based materials, (non-metal) pigments and polymers will often be less clear and the solute may, for example, depend on the degree of hydroxylation. It is therefore necessary to determine the solute in relevant media and conditions to be clear if and to which solute read-across can be performed.

Other groups indicated by Arts *et al.* [2] are “NMs with a High Aspect Ratio (HAR)”, “passive NMs”, and “active NMs”. As indicated, these groupings focus on human health and can help to highlight the information needs for a specific NM or a group of NMs. For example, NMs that fall into the group with an HAR would most likely induce toxic effects due to their morphology, especially for rigid, biopersistent HAR materials and exposure via inhalation [16].

The group “passive NMs” relates to NMs that are biopersistent, non-fibrous NMs which do not have surface reactivity and do not elicit a specific cellular effect and do not prevail in biological fluids in a well-dispersed form [2]. Criteria proposed by these authors to assign a NM to this group include the absence of toxic component(s) (less than 0.1% by weight), low surface reactivity (<10% of Mn_2_O_3_ in specific assays), an average agglomeration number ≥3, no cellular effects at ≤10 µg/cm^2^ and a confirmatory threshold by for example an No Observed Adverse Effect Concentration (NOAEC) (>10 mg/m^3^) in a short-term inhalation study. The ECETOC concludes that passive NMs are considered to possess no or a very low hazard potential, and suggests using a threshold value (a No Observed Adverse Effect Concentration NOAEC) of a benchmark passive NM for this group, at least for inhalation [2]. In the present paper, it is proposed that issues of special attention for the “passive NMs” are at least the distribution to target organs, potential accumulation and long-term effects, and for environment biopersistence and trophic transfer. Subsequently, safety has to be demonstrated either by testing or read-across as indicated here under “application of read-across”.

The group “active NMs” relate to NMs with specific toxic constituents and is determined according to Arts *et al.* [2] by chemical composition, dissolution rate, surface reactivity, dispersibility and/or cellular effect. ECETOC also proposes that NMs in this group undergo further subgrouping and/or require additional testing [2]. Further subgrouping can relate to mechanistic toxicological pathways as proposed by Nel *et al.* [15]: Redox activity and Reactive Oxygen Species (ROS) formation, dissolution, shedding toxic ions, cationic toxicity, inflammasome activation, photoactivation, and membrane lysis. The authors of the present paper think that special attention for active NMs issues should be given to effects related to the mechanistic toxicological pathway as well as distribution to target organs, potential accumulation and long-term effects, biopersistency and trophic transfer similar to the “passive NMs” group.

Grouping based on exposure can also steer the risk assessment process. This kind of grouping could be based on similar applications and/or processes e.g., the packaging process for a powder, spraying a solution, as well as physical form. This kind of information, as for example gathered in the MARINA exposure scenario library for NMs [21] is, as indicated before, relevant for RES identification. Also information on physicochemical properties during various life cycle stages [25] and information on release during specific activities or weathering from similar materials can be informative for RES determination. For example, agglomeration may reduce the transportation across biological barriers such as gastrointestinal tract [26]. Via this kind of grouping, information on exposure for similar NMs can at present be used in the risk assessment process.

### 6.3. Ad Five: Scientifically Justified Read-Across for a Specific Endpoint or Test

When applying read-across (which is always end-point specific and applies to hazard information), information on one or more endpoints relevant for risk assessment of a specific NM or of a group of NMs (target material(s)) is predicted by using available data from another NM or group of NMs (source material(s)). This means that testing of the target NM or group of target NMs is not required if a substantiated justification of why data from the source material(s) are applicable to the target material(s) can be provided. For non-nano substances, the ECHA guidance prescribes structural similarity between the target and source substance(s) as needed for read-across, for example related to common functional groups [10]. The understanding of structural similarity may be relevant for NMs but clearly needs further interpretation. Here, we discuss under which circumstances and how information from one NM can be used for another NM, both for NMs of the same chemical composition and for NMs of different chemical compositions. In addition in some cases, e.g., for quickly dissolving NMs, a non-nano (bulk) substance can be used as source material.

When it is clear at the end of Phase 1 of the MARINA RAS or from regulatory requirements which hazard endpoint(s) need(s) to be addressed, read-across among NMs or from bulk materials becomes an option. In other words, it can be considered if testing for that specific hazard endpoint is required for the NM under investigation, or if it is possible to use data from another material. As for chemicals in general, the first step is to identify potential source materials and provide a hypothesis to justify the read-across, which are obviously closely connected processes as the use of the source material should fit into the justification. Preconditions for read-across are:

- Information on at least chemical and physical properties and intended use of the target material(s) is available.
- Information on at least chemical and physical properties of the source material is available.
- Hazard/toxicokinetic data of the source material relevant to the endpoint is available.
- The quality of the physicochemical and hazard/toxicokinetic data of the source material is high and a suitable test procedure is used and described in sufficient detail.
- The source materials are appropriate for the justification for read-across.

Justification for read-across will probably be more easy when the physicochemical similarity between source and target materials increases. In general, source and target materials will be of the same group (quickly dissolving NMs, biopersistent HAR NMs, passive NMs, active NMs) and often of the same chemical composition and with a similar production process. Note that, in principle, a different justification is possible for different source materials.

As there is a lack of experience on route-to-route extrapolation for NMs and, in general, route-to-route extrapolation also for chemicals is associated with potential difficulties, it is considered that for NMs, for the time being, data from a source material can only be used in case of the same exposure route for source and target material. For example, NM behaviour can be route-dependent, e.g., due to differences in protein binding [27]. In addition, the exposure route is usually related to different exposure conditions (exposure medium, duration) and physicochemical properties that can affect toxicokinetics and hazard. The MARINA RAS already takes into account that physicochemical properties may change during the life cycle, and the information gathering aims at covering the physicochemical properties of the NM in the RES [8].

The data to be used for read-across should be high quality and generated according to a suitable test procedure, which is preferably assessed by using a scientifically founded procedure or guidance. The OECD-WPMN (Working Party on Manufactured Nanomaterials) concluded that in general the OECD test guidelines are applicable to NMs, and that special attention is needed for the sample preparation and dispersion [28,29,30]. In addition, several EU funded projects are undertaking effort to ensure data quality. For example, the NANoREG project (http://nanoreg.eu/) is making major efforts to develop guidance and procedures to obtain high quality data for NMs in a harmonised manner to facilitate comparisons. These insights can also help to assess the quality of reported data.

The first possibility to justify read-across is to identify source materials, which have mostly similar physicochemical properties and which can be ordered so that the values of the physicochemical properties of the target material are encompassed by values of the correspondent physicochemical properties of source materials. Hence, target and source materials should be very similar with regard to physicochemical properties and the number of available data and the ordering of the variable physicochemical property allows for interpolation or use of available data from the worst case source material. A substantiated justification should be provided that explains the effect of the variable physicochemical property on the endpoint. As several physicochemical properties may vary at the same time, it is expected that suitable source materials for NMs rarely exist.

**Figure 5:**
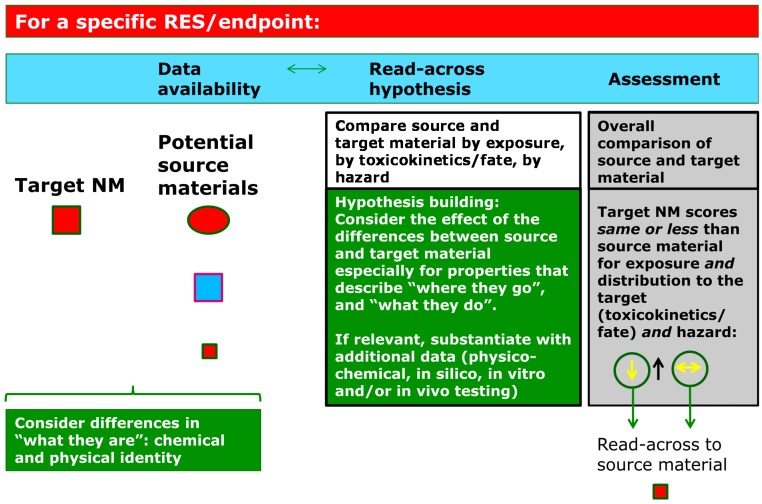
Considerations for a justification of read-across among NMs for a specific relevant exposure route (RES) or endpoint. The differences in chemical and physical identity between target and potential source material(s) are considered in relation to their relevance for affecting exposure, toxicokinetics/fate and hazard. Hypothesis development for read-across justification and identification of potential source materials are related processes. The justification can be substantiated by argumentation that exposure to the target material is very similar or less than for the source material(s) (exposure element), and that a very similar or smaller amount of the target material reaches the target site (toxicokinetics/fate element), and the target material is equally or less hazardous than the source material (hazard element). This justification needs to be scientifically correct and can be based on available knowledge on relationships between physicochemical properties and exposure, toxicokinetics/fate and hazard and may be complemented with physicochemical data, in silico, *in vitro* or if needed *in vivo* data.

Another possibility to substantiate justification for read-across is that the identified differences in physicochemical properties between source and target material(s) are systematically described and assessed, as is also suggested by Walser and Studer [31]. First, the available information on the relationships between the physicochemical properties that differ between target and source material(s), and exposure, toxicokinetics/fate and hazard relevant for the endpoint under investigation is considered and elaborated upon (see Figure 5). This elaboration relates to the properties that describe “Where they go” Fundamental behaviour, and “What they do” Reactivity as presented in Figure 1. It may be necessary to provide additional information to substantiate these relationships, for example by additional data on physicochemical properties of the source and/or target material(s), Quantitative Structure Activity Relationships (QSAR) (once available for NMs) [7], general relationships between physicochemical properties and exposure, toxicokinetics or hazard, or *in vitro* or if needed “limited” *in vivo* testing. Finally, for a specific endpoint an overall assessment on the applicability of data from the source material(s) to the target material(s) is performed (see Figure 5). For the overall assessment, a conservative approach is proposed for the time being by requiring that it can be justified that exposure, toxicokinetics/fate and hazard are very similar or worst case for the source material compared to the target material. In that case, data from the source material can be used for the endpoint or test under consideration for the target material. The concept of applying read-across to a worst case source material is similar to the “worst case approach” mentioned in the recently published Read-Across Assessment Framework (RAAF) by ECHA [32]. ECHA here indicates that read-across can be applied if the strength of the effect(s) of the target substance can be expected to be lower than the strength of the effects of the source substance. ECHA suggest that scientific explanations for such situations can be based on kinetic considerations and/or potency considerations.

A few examples of how to substantiate the justification are provided here for illustration. Argumentation for the exposure element of the justification may for example be based upon the expected release or level of exposure, or physicochemical information on the degree of aggregation/agglomeration at relevant conditions. For human health, the toxicokinetic element of the argumentation may be substantiated by *in vitro* or (limited) *in vivo* information on translocation across the portal of entry, distribution to and clearance from tissues, or dissolution in macrophage fluid. For the environment, the fate element of the argumentation can be substantiated with information on release in environmental media (water, sediment, soil, air), or aggregation/agglomeration behaviour or complexation behaviour with environmental constituents in these media. The hazard element of the argumentation may be substantiated firstly by *in silico* or *in vitro* data, and, if needed *in vivo* data, which may be included to assure the same mode-of-action and to compare the hazard potency between source and target material.

It should be noted that effect(s) of the differences in physicochemical properties between target and source material(s) should be considered separately for exposure, toxicokinetics and hazard, due to the current limited understanding of the behaviour of NMs, as illustrated by Figure 2 (on life cycle and biological pathways). Obviously, the proposed worst-case approach on exposure and toxicokinetics/fate and hazard may be reconsidered on a case-by-case basis depending on the situation, and should be accompanied with a scientifically sound argumentation. Note that the existing argumentation on structural similarity, required for read-across of chemical substances (e.g., OECD [9], ECHA [10]) is transformed here for NMs into an argument on relationships between the variable physicochemical property/ies and exposure, fate/toxicokinetics and hazard. Similar to non-nano substances in a regulatory context, toxicokinetic information is considered a key element in the argumentation [10].

It should also be noted that in the present approach for read-across, it is implicitly assumed that the effect under consideration is the same for source and target material. As another type of effect may be more critical for the target material, this may require some consideration and discussion in the justification. Similarly, differences in uptake mechanism (passive *vs.* active) between target and source material may also need further consideration, for example as non-linear uptake often occurs for active mechanisms at increasing concentrations.

If read-across options seem to be scientifically debatable or time and/or cost intensive, actual testing of the target material may be a better option.

## 7. Final Considerations and Future Developments

The presented approaches for grouping and read-across pave the way for making best use of available knowledge on tested nano and non-nano materials to obtain information needed for risk assessment of one or more (toxicologically) untested or unassessed NMs. To this end, grouping of NMs can be used for different reasons, and these are: (i) to acquire information on one or more RESs or endpoints via a targeted testing strategy whose results cover the entire group of NMs, (ii) to identify generic groups based on information on similar materials to guide the identification of further information needs and indicate possibilities for read-across early in the risk assessment process, (iii) to come to a scientifically founded argumentation for read-across to a target NMs for a specific endpoint by using data from source material(s). The generic grouping approaches allow for further subgrouping based upon increased knowledge on the relationships between physicochemical properties and exposure, fate/toxicokinetics and (eco)toxicity. A nanomaterial may belong to more than one group, e.g., if a NM is quickly dissolving and with an HAR, or related to the different applications of groupings and read-across. For example, an NM can be allocated to a group based on similar physicochemical properties to design a testing strategy whose results cover the entire group (ad 1 in Figure 4). Subsequently, in Phase 1 of the RAS information, similar materials are used to highlight information needs on hazard and exposure in the data gathering process (ads 3 and 4 in Figure 4, respectively), and, finally, for specific endpoints in Phase 2 of the RAS, read-across to data of specific source materials is considered (ad 5 in Figure 4).

It should be noted that the presented overview of approaches for grouping and possibilities for application in the MARINA RAS are discussed at a rather abstract level. In order to increase the robustness, practical application, *i.e.*, via case studies is essential. Another important issue that still needs to be addressed is how to deal with uncertainty in the risk assessment, grouping and read-across process. More and more developments are ongoing to include information on uncertainty as for example in an approach laid down by Gajewicz *et al.* [33] for preliminary hazard assessment and hazard prediction. Furthermore, details on decision making should be developed. These issues will be taken up in the future.

The overview clearly shows that information on a number of physicochemical properties of an NM should be available to start the information gathering process and to consider options for grouping and read-across. Without this information, these processes cannot be initiated.

Furthermore, it is acknowledged that building and substantiating a scientifically founded read-across argumentation is in most cases a work-intensive process, though it is expected to become easier and be more successful as more high quality data of NMs, *i.e.*, more potential source materials, become publicly available and as knowledge on relationships between NM properties and exposure, toxicokinetics and hazard increases. Better understanding on the behaviour of NMs further enables the development of more *in silico* tools and simple tests that can be used to substantiate the exposure, toxicokinetic or hazard element of the justification. For example, *in vitro* tests for a specific mechanistic injury pathway as suggested by Nel *et al.* [15] can be developed to obtain information for the hazard element of the read-across argumentation for human health. A simple test like dissolution in macrophage fluid may be used to substantiate that a source material accumulates to a smaller extent in a target site within a living organism than a target material. Development and steps towards acceptance of such simple tests including description of their applicability is highly recommended.

In conclusion, grouping and read-across can be valuable tools to limit the amount of testing for risk assessment of nanomaterials and at the same time meet the prerequisite to obtain sufficient information to assess the safety. Different applications for grouping and read-across of nanomaterials can be distinguished and have been presented within the broader perspective of the MARINA Risk Assessment Strategy. These approaches pave the way for better use of available information on nanomaterials and are flexible enough to allow future adaptations related to scientific developments.
